# Translating insights into tumor evolution to clinical practice: promises and challenges

**DOI:** 10.1186/s13073-019-0632-z

**Published:** 2019-03-29

**Authors:** Matthew W. Fittall, Peter Van Loo

**Affiliations:** 1 The Francis Crick Institute, 1 Midland Road, London, NW1 1AT UK; 2 University College London Cancer Institute, 72 Huntley Street, London, WC1E 6DD UK; 3 Wellcome Trust Sanger Institute, Wellcome Genome Campus, Hinxton, Cambridgeshire, CB10 1SA UK; 4 University of Leuven, Herestraat 49, B-3000 Leuven, Belgium

## Abstract

Accelerating technological advances have allowed the widespread genomic profiling of tumors. As yet, however, the vast catalogues of mutations that have been identified have made only a modest impact on clinical medicine. Massively parallel sequencing has informed our understanding of the genetic evolution and heterogeneity of cancers, allowing us to place these mutational catalogues into a meaningful context. Here, we review the methods used to measure tumor evolution and heterogeneity, and the potential and challenges for translating the insights gained to achieve clinical impact for cancer therapy, monitoring, early detection, risk stratification, and prevention. We discuss how tumor evolution can guide cancer therapy by targeting clonal and subclonal mutations both individually and in combination. Circulating tumor DNA and circulating tumor cells can be leveraged for monitoring the efficacy of therapy and for tracking the emergence of resistant subclones. The evolutionary history of tumors can be deduced for late-stage cancers, either directly by sampling precursor lesions or by leveraging computational approaches to infer the timing of driver events. This approach can identify recurrent early driver mutations that represent promising avenues for future early detection strategies. Emerging evidence suggests that mutational processes and complex clonal dynamics are active even in normal development and aging. This will make discriminating developing malignant neoplasms from normal aging cell lineages a challenge. Furthermore, insight into signatures of mutational processes that are active early in tumor evolution may allow the development of cancer-prevention approaches. Research and clinical studies that incorporate an appreciation of the complex evolutionary patterns in tumors will not only produce more meaningful genomic data, but also better exploit the vulnerabilities of cancer, resulting in improved treatment outcomes.

## Background

Over time, the therapeutic approach to cancer is evolving from targeting the clinical phenotype (tumor size, location, stage, histological type, and grade), to targeting a molecular phenotype (such as surface receptor status or the presence of activating or sensitizing mutations) [1, 2]. The clinical phenotype can be targeted spatially with surgery and radiotherapy or systemically using cytotoxic chemotherapies. The molecular phenotype has been targeted by both direct and indirect endocrine manipulation, by an array of small molecule inhibitors, and by monoclonal antibody therapies. Both approaches typically consider the target to be static (to be treated until clinical failure) and homogeneous (one sample represents all tumor cells).

The application of evolutionary concepts to cancer was proposed several decades ago by Peter Nowell [3]. Reliable exploration of the degree of variation within and between cancers has only become possible with the increasing availability of next generation sequencing and associated computational analysis [4–6].

All of the cells within a tumor are unique, comprising different somatic variants and epigenetic and transcriptomic states. Even normal cells are likely to accrue approximately three somatic mutations every cell cycle [7, 8]. Most of these changes will have no functional impact and are ‘passengers’ on the cells’ evolutionary journey (Box 1). Somatic mutations (or epigenetic changes) that have an advantageous functional impact are ‘drivers’ and will allow a cell to expand clonally and outcompete its neighbors. When a clonal expansion goes to completion, the entire population will be ‘clonally’ descended from that founder cell, or clone. The last complete clonal expansion will have arisen from the most recent common ancestor (MRCA), defined as the most recent individual cell from which all existing cancer cells in a cancer sample are descendants. If a clonal expansion or sweep is incomplete, the expanded population is subclonal, comprising only a fraction of the tumor cells. Diverging subclones with mutually exclusive mutations can co-exist within a tumor [9]. Intra-tumor heterogeneity, or the presence of subclones possessing private mutations within a tumor, has been observed across many cancer types and seems to be nearly ubiquitous [10, 11].

The dynamics of evolution in cancer are still not fully understood [12]. Traditionally, mutation and selection are thought to be slow iterative processes that occur throughout a cancer’s lifetime, a process of gradual evolution. The patterns of mutations observed in some tumors, however, suggest that mutations can also be acquired in sudden bursts, leading to punctuated evolutionary steps [13–19].

An emerging wealth of cancer genome sequencing data is informing our understanding of tumor evolution, and will cause a fundamental paradigm shift in our approach to cancer. This will impact all aspects of cancer management, including cancer therapy, monitoring, early detection, and prevention (Table 1).

**Table 1:** Promises and challenges in translating insights into tumor evolution to clinical practice

	Therapy	Monitoring	Early diagnosis and stratification	Prevention
Promises	• Clonal therapy targeting clonal mutations to eradicate all tumor cells (such as targeted therapy or immunotherapy) • Preempt resistance • Adaptive therapy to chronically control disease	• Bespoke monitoring based on tumor-specific mutations	• Identify genetic changes meriting intervention	• Mutational signatures can suggest etiological factors that drive early tumorigenesis
Challenges	• Sampling strategy • Inevitable clonal monotherapy resistance • Bespoke combination therapies complicate toxicity and licensing	• High cost • Novel mutations or subclones may be missed • Early detection of relapse may not improve outcome	• Normal tissues contain canonical cancer mutations • Early diagnosis may not improve outcome	• Exogenous factors may not be preventable • Some tumors may not be preventable (such as those of children or young adults)

## Measuring intra-tumor heterogeneity and tumor evolution

Implicit in the heterogeneity of tumor cells and essential for evolution is variation in either the genome or the epigenome [20–22]. Although epigenetic heterogeneity has been shown to have prognostic utility [23–26] and is the subject of intense study, genetic heterogeneity is better understood at present, and is the focus of this review.

**Fig. 1:**
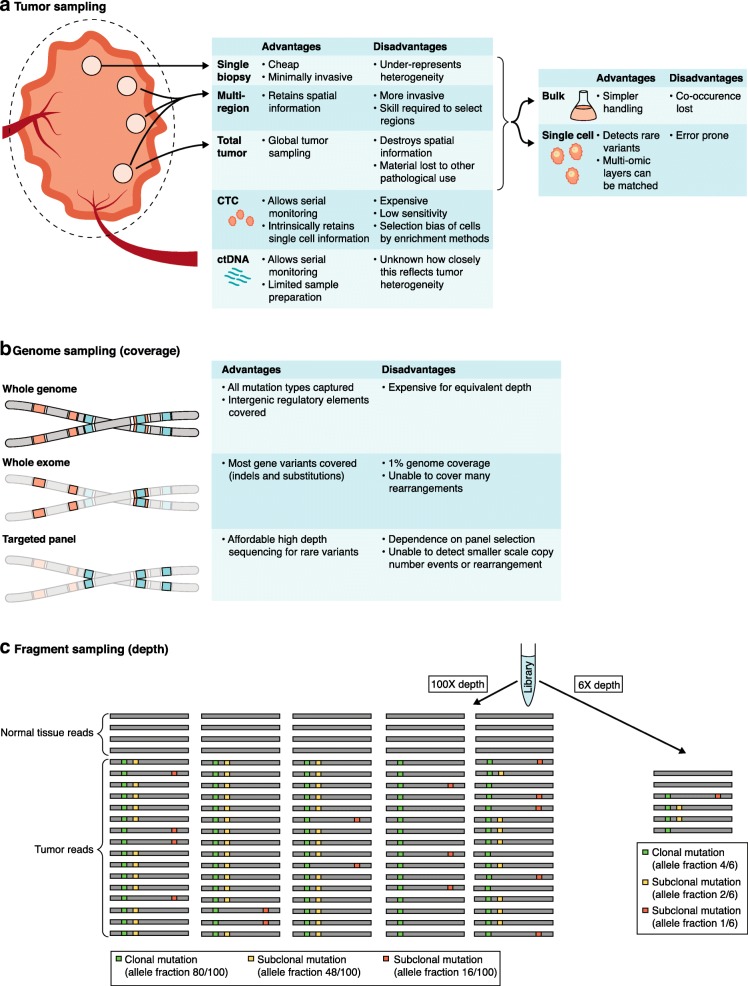
Sampling decisions required for comprehensive and evolutionary description of tumors. Tumor genomic sampling can be considered to fall into three separate domains. **a** Sampling of tumor material, either directly from a tumor mass or shed into the circulation. Samples from the tumor mass can either be pooled as a bulk specimen or disaggregated into single cells. **b** Only portions of genomic material are sampled and assessed; either targeted panels of a few hundred genes can be used or the whole exome or whole genome can be profiled. **c** Bulk DNA extractions may contain millions of DNA molecules. These are contributed by different parental alleles from both tumor and normal cells. Samples frequently contain 10–80% normal cells. Library preparation and sequencing only samples a tiny fraction of the available DNA fragments. The schematic shows a representation of sampling at two different sequencing depths (100X and 6X) and illustrates how higher sequencing depths allow more accurate determinations of the frequencies of specific mutations and their clonal or subclonal status. *ctDNA* circulating tumor DNA

Intra-tumor heterogeneity and evolution can be inferred from the pattern of mutations that is detected. Clonal mutations, which are common to all cells within a tumor, were present in the tumor cells’ most recent common ancestor, whereas subclonal mutations were acquired later and are therefore only found in a proportion of tumor cells (Box 1). The frequency of a mutation in sequencing data (the variant allele frequency (VAF)) can be used to establish its clonality. VAF is influenced by both the proportion of cells that possess the mutation and the number of both mutated and un-mutated copies of that DNA locus. Mutation frequencies can be estimated by sampling, which has intrinsic spatial, genomic, and statistical limitations (Fig. 1). Intra-tumor heterogeneity has been extensively explored using exome or genome sequencing of multiple regions of resected primary tumors [9, 12, 19, 27–29]. Paired primary–metastasis studies and post-mortem studies have allowed detailed insight into the evolution and patterns of spread of metastases [30–33]. Intra-tumor heterogeneity has been shown to be prognostic across cancer types [10, 34, 35], and is predominantly associated with the degree and heterogeneity of aneuploidy. It has also been shown to impact therapy: potentially targetable driver mutations can be subclonal, suggesting that treatment would only be partially effective [36].

Describing tumor evolution requires measurement over time. Models of tumorigenesis, such as the ‘Vogelgram’, were created by sampling different stages of cancer progression across a population [37, 38]. The initial Vogelgram in colorectal cancer was established by probing a limited number of putative oncogenes, identified from hereditary cases, across the histologically defined spectrum of disease [39]. Mutations that are found across different stages of disease are assumed to arise early in tumor evolution, whereas those found only in established invasive cancers can be assumed to occur later in tumor evolution. Rarely, in individuals with predisposing risk factors such as those who have colitis-associated colon cancer, the whole spectrum of tumor progression can be observed simultaneously [27]. Despite applying modern genomic techniques, models of progression can remain elusive if the genome is already markedly aberrated in pre-invasive lesions, as in the precursors of lung squamous cell carcinoma [40]. This modeling approach also relies on the assumption that cancers of the same histology have a highly stereotyped genetic progression that is common to different tumors.

Computational approaches have been developed to infer the history of an individual tumor that is already established from its own genome, as recently reviewed [41, 42]. Although these approaches typically allow only partial reconstruction of a tumor’s evolutionary history, from a single biopsy, aggregating results across multiple tumors can be a powerful approach [42]. Taking multiple samples from the same tumor over time or across space can also significantly increase the power of these reconstruction approaches [41, 42]. In metastatic solid organ tumors, repeated sampling over time is challenging, so hematological malignancies have been studied most extensively in this context [43–46]. Circulating tumor DNA (ctDNA) and cells shed from solid tumors offer the potential to track subclonal mutations, albeit with limited sensitivity and specificity.

Most DNA sequencing has been performed on pooled DNA from multiple cells and, consequently, ambiguity can remain as to whether mutations co-occur in the same cell. Single-cell sequencing can overcome this, albeit at higher cost and at the expense of substantial sequencing artifacts [47–50]. High-throughput techniques have been developed for analyzing large numbers of single cells, although these methods are most advanced for transcriptome sequencing [51]. Single-cell sequencing of other ‘omic layers is currently relatively costly and available for fewer cells [52], but exciting high-throughput approaches are now emerging [53]. Techniques to analyze multiple layers simultaneously have also been developed recently [54–56], but these are currently costly and lower throughput. These ‘multi-omic’ approaches are likely to significantly improve the interpretation of non-genetic cellular heterogeneity. Such interpretation is also confounded by heterogeneity among non-tumor cells that results from the variety of cell types and states within a tumor [57, 58].

Future approaches for measuring tumor heterogeneity that could be used clinically would need to satisfy the following criteria: (i) sampling should be minimally invasive or performed as part of tumor resection; (ii) sampling of the tumor should be as comprehensive as possible, ideally without any spatial biases; (iii) sample handling and preservation will need to be simple and readily available in the clinic; (iv) simple proxy biomarkers need to be available to assay heterogeneity reliably; and (v) assays need to be rapid and cost-effective.

Recently, a conceptual consideration of how evolution and heterogeneity could be summarized was explored in a consensus statement by Maley et al. [59]. They proposed binary divisions of the degree of heterogeneity (diversity, D) and evolution (rate of change, ∆) that could be combined in a single four-level Evo-Index. As yet, it is not clear how these scores would be generated or whether such a simple binary system is informative.

## Can tumor evolution guide cancer therapy?

The rational design of cancer therapies based on genomic data has to date, with a few notable exceptions, been expensive and has delivered limited benefit to patients [60]. Even therapies specifically targeting prevalent tumor mutations, such as the *BRAF* V600E mutation in melanoma [61] and a variety of *EGFR* point mutations in lung cancer [62], only lead to relatively short-lived tumor responses. Understanding the heterogeneity that exists within tumors and their ability to evolve in response to therapy may allow more optimized treatment strategies (Table 1).

### Individual clonal therapies

The simplest conceivable therapeutic approach is to target individual clonal mutations. By targeting mutations that are present in all tumor cells, the entire tumor could in theory be eradicated. Previous targeted therapies have, to some degree, implicitly relied on the presumption that mutations that are highly prevalent in different tumors are probably early events in tumorigenesis and therefore likely to be clonal.

**Fig. 2:**
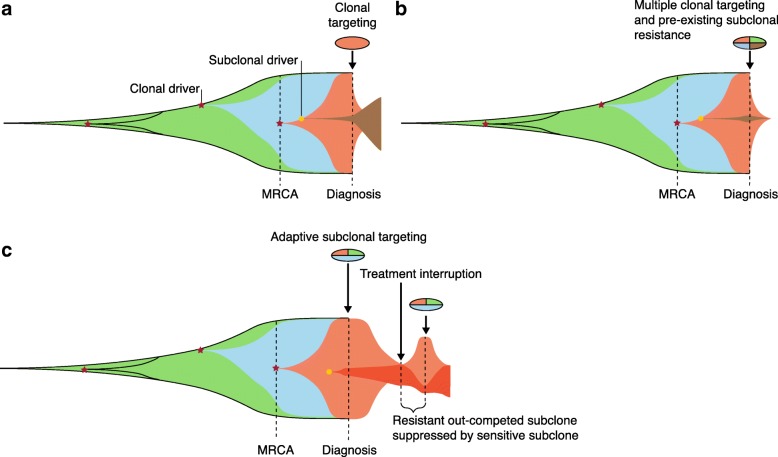
Evolutionary therapy strategies. Schematics of tumor populations in which each different color implies a new subclonal population. Therapies are denoted by *segmented ovals*, in which the targeted populations are indicated by the segment shading. **a** Targeting a clonal mutation that developed in or prior to the most recent common ancestor (*MRCA*). Resistance may emerge because a (rare) subclone with intrinsic resistance to that therapy (for example, an *ESR1*-activating mutation) existed prior to therapy. **b** Targeting of multiple drivers is more likely to lead to tumor extinction. **c** In adaptive therapy, treatment is discontinued before sensitive cells (*pink*) are eliminated, allowing them to grow back and suppress resistant cells (*red*). The resistant subclone would be expected to have an intrinsic survival disadvantage that is related to its resistant phenotype, for example, it may have lost the targeted driver mutation

In most cases, single clonal mutations, which are thought to be functionally relevant driver mutations, have been targeted directly. In established cancers, this invariably results in the acquisition of treatment resistance. The simplest examples are the resistance to endocrine therapy in metastatic breast and prostate cancer. The mechanisms of these resistance phenomena are now relatively well understood. Many breast cancers depend on estrogen signaling and are initially sensitive to therapies that reduce the level of circulating estrogen or that target the cellular estrogen receptor, such as aromatase inhibitors or selective estrogen receptor modulators, respectively. Treatment resistance frequently arises when tumor cells develop constitutive activity in the estrogen receptor through mutation of its gene, *ESR1* [63]. Likewise, prostate cancers are almost ubiquitously driven by androgen signaling, sensitizing them to chemical or surgical castration. Prostate cancer cells compensate for medically depleted circulating androgen levels through a number of different mechanisms, including amplification of the androgen receptor [64]. Gundem et al. [31] demonstrated that multiple separate tumor cell populations, across distinct metastatic sites, can develop unique androgen receptor amplifications—a demonstration of convergent evolution. The widespread evolution of resistance suggests that clonal monotherapies are unlikely to achieve permanent tumor control or cure. For those with slow-paced advanced disease, or those who would not tolerate more intensive therapy, individual therapies will continue to play an important role. Most responses to targeted therapies, however, are both incomplete and short-lived and require improvement (Fig. 2a).

Even when a mutation is not treated directly, tumors can develop resistance. Synthetic lethality is a treatment approach that exploits a cellular vulnerability exposed by a clonal driver mutation. *BRCA* mutations in breast and ovarian cancer, both inherited or acquired, increase genomic instability due to disruption of the repair of double-strand DNA breaks, which not only produces variation during tumorigenesis but also increases the reliance of these tumors on other DNA-repair mechanisms. This is exploited for therapy by inhibiting the single-stranded DNA repair PARP enzymes [65, 66]. PARP inhibition causes the accumulation of lethal DNA damage specifically in tumor cells. *BRCA* mutations can, however, undergo somatic reversal in multiple tumor subclones, leading to resistance to PARP inhibition [67–69].

Resistance to therapy typically results from mutations, which may pre-exist or can appear subsequent to the therapy, or from non-genetic factors. Mutations that exist prior to treatment exposure might be rare, and therefore undetectable by present assays. Once treatment creates selective pressure, resistant cells carrying these mutations will persist and become apparent. It is possible, and perhaps likely in larger tumors, that most resistance mutations exist prior to therapy exposure, even for conventional cytotoxic therapies [52]. Resistance mutations may also occur de novo after treatment exposure, perhaps having been induced by iatrogenic mutagenesis [70, 71]. Unless these mutations are of a distinct type, known to be induced by therapy, it is difficult to exclude the possibility that they did not exist prior to treatment at a very low and undetectable frequency. Resistance may also be non-genetic and either related to cell state plasticity or to a specific molecular resistance pathway, such as aurora kinase activation in anti-EGFR-treated lung cancer [72, 73], but further understanding of these non-heritable resistance mechanisms is needed. There are broad principles of treatment resistance that are common between cancer and infectious diseases [74]: like tumor cell populations, pathogens can be also be genetically heterogeneous [75], and as observed in the examples of HIV and *Mycobacterium tuberculosis*, they rarely have prolonged responses to monotherapy.

In principle, individual clonal therapies may still be used curatively if employed very early in tumor evolution, as proposed by Mitchell et al. [76]. Clear cell kidney cancers were modeled to have deleted *VHL* (on chromosome 3p) several decades prior to a second mutational hit to the remaining *VHL* allele. Proliferation and tumorigenesis only accelerate after both alleles of *VHL* are mutated. Therefore, the pool of mutated cells is probably only a few hundred cells for a prolonged period. Depleting this small cell population—even marginally—with a therapy, sensitized by 3p loss, would reduce the probability of a cell with a second hit mutation ever arising. This would have to be achieved decades before these cells become detectable, and therefore would most likely involve the preemptive treatment of healthy individuals. However, considering that most tissues may harbor equivalent cell populations [8, 77, 78], such prophylactic management may not be clinically, economically, or ethically feasible.

### Combined clonal therapies

Predictions of a tumor’s evolutionary response to a therapy can allow pre-emptive measures to prevent resistance. For example, ABL1 inhibition in chronic myeloid leukemia (CML), characterized by clonal *BCR*–*ABL1* fusions, has revolutionized therapy for this disease, yet the development of resistance remains a challenge in a proportion of patients. Combining different classes of ABL1 inhibitors with mutually exclusive profiles of resistance mutations can preempt the emergence of resistant subclones (Fig. 2b). Preclinical application of this approach has resulted in durable responses [79].

Combining different clonal therapies might also reduce the emergence of resistance. Many breast cancers are thought to have cell-cycle dysregulation related to the cyclin-CDK-Rb pathway, in addition to estrogen sensitivity [80]. The addition of CDK4/6 inhibition to aromatase inhibition does indeed prolong the response in patients with metastatic disease. This delays the need for conventional cytotoxic therapy, but at the price of increased toxicity compared to endocrine therapy alone [81].

The development of effective combination therapies requires a comprehensive understanding of mutation clonality and resistance mechanisms. Metastatic melanomas frequently have activating mutations in the MAPK pathway, and resistance to BRAF inhibitors was thought to result from downstream MEK activation [82, 83]. Trials combining MEK and BRAF inhibition in melanoma have demonstrated modest clinical benefit [84, 85]; however, resistant tumors often have multiple different detectable MAPK mutations, suggesting convergent evolution [86].

Ideally, larger numbers of drivers could be targeted simultaneously or sequentially, depending on the pace and nature of the evolutionary response of the tumor. Such combination therapies will impact toxicity management, although not always detrimentally. In fact, the addition of MEK inhibition to BRAF inhibitors reduces the cutaneous side-effects that are associated with BRAF inhibitors. The toxicities resulting from combination treatments may, however, require complex pharmacological adjustments that have implications for trial design, drug licensing and healthcare economic assessments.

Many tumors have only few clonal driver mutations and will require alternative strategies [87–89]. Effective pharmacological options for targeting driver mutations are also relatively limited. Some driver mutations may be treatable indirectly, either by collateral lethality, whereby susceptibilities created by the loss of genes adjacent to deleted tumor suppressors are harnessed, or by synthetic lethality [90, 91]. Alternatively, immunotherapy exploits the antigenicity of mutations, regardless of their driver status and without relying on the recurrence of mutations in different patients. Where durable clinical responses to immunotherapy have been seen, they are probably brought about by the simultaneous targeting of multiple clonal mutations. Indeed, one of the potential predictive markers of response to immune checkpoint blockade in non-small-cell lung cancer and melanoma is the clonal neoantigen load [92, 93]. If a common mechanism of resistance to an immunotherapy can occur, (epi) genetic variation and selection could drive tumors towards it, even when multi-pronged approaches are used. These mechanisms of immune editing are still a subject of intense study. They include an ability of tumors to reduce their antigen-presenting capability. In melanoma, lung, and ovarian cancer, these changes have been shown to result in part from either somatic (often subclonal) or germline loss of heterozygosity of the HLA locus [94–96]. Equivalent loss of expression of class II MHC may also result in treatment failure after allogeneic bone marrow transplant for acute myeloid leukemia [97]. Without a full and diverse HLA repertoire, many neoantigens cannot be successfully presented on the surface of tumor cells and therefore are not recognized by an adaptive immune response.

### Targeting subclonal mutations

The detection of subclonal mutations is still an active research topic and therefore potential strategies for their therapeutic use are only conceptual at present. The simplest approach is to target a combination of multiple subclonal mutations, probably coupled with a clonal therapy. In rare circumstances, such as those recently suggested in pediatric brain tumors, subclonal populations might be highly functionally interdependent [98]. In these circumstances, even subclonal population depletion might have a profound effect on the tumor as a whole. Alternatively, if the relative importance and the clinical impact of different subclonal populations can be measured, then those causing the greatest symptomatic burden could be prioritized. Implicit in this more strategic approach is the acceptance that other cell populations that cause lower symptomatic burden will not be eradicated, representing a shift to managing cancer as a chronic disease without the intent to cure [99].

A combination of conventional cross-sectional imaging with the monitoring of circulating markers could be used to identify spatially or mutationally distinct metastases. If lesions are spatially segregated, they may be amenable to local therapies: surgery, cryotherapy, focused ultrasound, or stereotactic radiotherapy. If they are characterized by treatable mutations, additional systemic therapies could be used. At present, proofs of this concept are yet to emerge.

Finally, the concept of adaptive therapy has also been proposed [100, 101]. Each of the subclones present in a tumor may be either sensitive or insensitive to a potential therapy. They compete for survival within the tumor environment and a mutation that confers resistance to a treatment, possibly through the loss or alteration of an oncogenic driver, might result in a growth disadvantage when that treatment agent is not present. With an adaptive approach, sensitive subclones can be treated to the point at which tumor size is reduced or growth is suppressed to achieve symptomatic benefit. Response may conceivably be monitored with a non-invasive surrogate biomarker, such as serum prostate-specific antigen (PSA) in prostate cancer. Thereafter, treatment can be reduced or withdrawn to allow the competitive suppression of resistant subclones (Fig. 2c). This approach is currently under evaluation in metastatic prostate cancer with the use of individualized PSA thresholds to guide the use of abiraterone, a CYP17A1 inhibitor [102]. To date, only small numbers of patients have been treated, albeit with good clinical outcome and reduced cumulative exposure to medication. It is worth noting that adaptive therapy is not the same as intermittent therapy, in which treatment may also be used discontinuously and with the monitoring of a biomarker, but without any individualization of treatment duration on the basis of response dynamics. For example, intermittent hormonal therapy has been attempted in prostate cancer. Crucially, trials such as TAP22 used fixed PSA thresholds rather than individualized thresholds [103, 104]. This could result in the depletion of treatment-sensitive clones, reducing their ability to suppress their treatment-resistant cousins.

## Therapy monitoring: circulating tumor DNA and circulating tumor cells

Liquid biopsies sample more readily available body fluids, mainly blood, for cellular or genomic material that has been shed from the tumor. They are heralded for reducing the invasiveness of clinical assays used for diagnosis [105, 106], prognosis [107, 108], molecular profiling [109], and response assessment [110–114]. Monitoring the treatment of more advanced disease may be substantially enhanced by monitoring the dynamics of different tumor cell populations.

The therapeutic approaches discussed above, particularly combination subclonal targeting and adaptive therapy, rely on accurate information about the relative importance of different subclonal populations in space and time. Liquid biopsies allow non-invasive assays that can easily be repeated over time. In particular, ctDNA is relatively stable and simple to handle, and its sequence content can be analyzed using a variety of approaches [115] (Fig. 1).

The detection of early subclinical relapse or minimal residual disease after attempted curative therapy has relied on detecting clonal mutations in circulation. Somatic structural variants are particularly amenable to highly disease-specific PCR-based approaches. Canonical disease-defining genomic rearrangements, such as the *BCR–ABL1* fusion in chronic myeloid leukemia, are routinely monitored in hematological malignancies to assess treatment response [116, 117]. Solid organ malignancies have fewer disease-defining rearrangements, but frequently possess unique somatic rearrangements that can be used to define bespoke monitoring panels [118, 119].

Monitoring of subclonal evolution has focused on evaluating somatic point mutations. Murtaza et al. [120] demonstrated that a dominant subclone, which was responsible for the progression of a chest wall breast cancer metastasis, was detectable by the increasing level of mutations private to that subclone. O’Leary et al. were able to use ctDNA in a small proportion of metastatic breast cancer patients, who were treated with the addition of the CDK4/6 inhibitor palbociclib, to both predict longer progression-free intervals [111] and detect emerging resistant subclones [121]. Furthermore, Abbosh et al. [30] showed that ctDNA was detectable 10–346 days (median 70 days) prior to clinical detection of relapsed lung cancer.

There are several challenges to the adoption of this approach. Clearly, bespoke ctDNA monitoring is costly. Abbosh et al. [30] estimated that even a limited bespoke monitoring panel, based on detected mutations from a single primary tumor region, would cost USD 1750 per patient. In addition, current analyses have only explored minimal numbers of detectable subclones and give an incomplete picture of their number and range. Whether there are substantial biases in the tumor cells that contribute circulating DNA is currently not known. It is likely that highly vascular and necrotic tumors will contribute more to ctDNA than tumors in cryptic sites, such as the central nervous system [122]. The use of other sources of cell-free DNA, such as stool [123], urine, cerebrospinal fluid, and effusions, may in part compensate for this [124, 125]. There are also likely to be genomic biases because cell-free DNA is predominantly thought to be generated by apoptotic nuclease activity which produces nucleosome-associated DNA fragments [126, 127], resulting in distinct chromatin-associated patterns. These patterns and the degree of apoptosis are likely to vary across tumor cell populations, and result in a bias in circulating tumor DNA.

The detection of subclonal mutations is also limited by the sensitivity of detection assays. Next-generation sequencing approaches that seek to gain an unbiased view of all detectable variants in circulation cannot identify rare subclonal mutations. In the Murtaza et al. [120] study, even clonal mutations had variant allele fractions of 3.8–34.9%. To compensate for this, most approaches, as exemplified by Abbosh et al. [30], use a specific amplification method based on fixed expected mutations that are detected in a sequenced primary tumor. This, by definition, means that de novo mutations that arose subsequent to the sampling of the primary tumor will not be detectable in circulation.

Circulating tumor cells (CTCs) can be analyzed using single-cell sequencing approaches. In a study by Carter et al. [128]*,* the copy number profile of circulating tumor cells at the time of diagnosis of small-cell lung cancer predicted the duration of response to chemotherapy. Cellular approaches are less likely to be confounded by the genomic aberrations that arise in other cells than the index tumor [129, 130]. As a result of the rarity of these tumor cells, they require significant enrichment which is likely to introduce biases, resulting in low sensitivity even for clonal tumor populations [131, 132]. Interestingly, Kwan et al. [133] demonstrated that after some initial filtration, an RNA expression-based signature can be used to detect breast cancer CTCs, and that the presence of these cells carried prognostic information in the setting of neoadjuvant chemotherapy.

## Can insight into tumor evolution improve early diagnosis, risk stratification, and cancer prevention?

In order to improve cancer outcomes, it is essential to alter tumor evolution. This can be achieved throughout the evolutionary timeline by preventing etiological factors, screening cell populations on the path to cancer, or stratifying cancers that will pose the greatest threat.

### Cancer screening

Cancer screening aims to reduce cancer mortality by increasing detection at a curable stage [134]. This needs to be carefully managed, however, as overtreatment of incidental findings causes unnecessary cost, harm, and anxiety [135]. This problem has beset the introduction of a prostate cancer screening strategy, as many low-grade prostate cancers can be managed with observation alone [136, 137]. Reliable predictive biomarkers of progression in detected lesions could increase the utility of screening programs. To date, risk stratification has relied almost exclusively on histological staging and grading.

Methods are being developed that recapitulate the early evolution of cancers using sequencing information from later-stage cancers alone, as recently reviewed [42]. In general terms, these methods utilize the number of copies of mutations on gained chromosomal segments to infer whether these mutations happened before or after that gain. For example, if a whole chromosome has been duplicated and there are two copies of a mutation found on that chromosome, then it is likely that the mutation occurred first and was duplicated with the chromosomal gain. By analysis of whole-genome sequencing data from primary and metastatic prostate cancers, Wedge et al. [138] have been able to retrospectively identify chromosomal changes that developed earlier in tumorigenesis. These findings, such as the early gain of chromosome 8q, recapitulated those previously found in prostate intraepithelial neoplasia (PIN), which is thought to be a precursor of prostate adenocarcinoma [139].

These approaches have also been applied to invasive cancers, which have less well characterized precursor lesions [76, 89]. Recently, the Pan-Cancer Analysis of Whole Genomes (PCAWG) initiative leveraged whole-genome sequencing data to infer evolutionary timelines across cancer types [140]. This work reproduced and refined classic models of mutational progression such as for colorectal cancer, in which *APC* mutations precede *KRAS* and *TP53* mutations. This information could define mutations that can be used to risk-stratify those pre-malignant or early invasive lesions that require intervention and those that do not. In addition, large datasets and novel computational methods [141, 142] may be able to detect stereotyped evolutionary patterns and trajectories in cancer evolution that may inform early diagnosis or risk-stratification approaches.

### Predicting tumor evolution—implications for risk stratification

A deeper and more comprehensive understanding of tumor evolution should allow us to understand how a cancer will behave in the future. This has specific implications for the risk stratification of established cancers. Incidental findings, such as small renal lesions that are often found during investigations for other conditions, are a clinical challenge because definitive resection is morbid but radiological and histological criteria are unreliable for prognostication [143]. In clear cell renal cell carcinoma, Turajlic et al. [87] have modeled that analyses of two biopsies can allow the quantification of intra-tumor copy number heterogeneity. This can discriminate lesions of higher and lower risk of progression, thereby potentially assisting in the decision-making process for small renal lesions. In a companion study, the same authors also suggested that richer information gleaned from more thorough tumor sampling can identify evolutionary profiles that are more likely to be associated with the development of metastatic disease [88]. In other cancer types, patterns of heterogeneity, such as copy number diversity in lung cancer [89] and pan-mutational diversity (so-called regional ‘explosions’) in childhood cancers [144], have also been shown to carry prognostic information. More transformative change to cancer prediction strategies will require the development of more complex computational tools and models [141, 142]. Much as weather forecasting models require vast amounts of measured data from the real world, cancer evolution models will require the sequence-based profiling of the evolution of many more cancers. Ultimately, this will allow these forecasts to guide the optimal management for each patient.

### Prevention of key early mutagenic processes

The identification of predisposing factors for cancer, whether heritable, environmental, or infectious, has previously relied on a combination of epidemiological and biological evidence. A deeper understanding of tumor evolution can lead to new insights into the impact of these factors on the genome.

Two clear examples of direct impact on the genome are ultraviolet (UV) radiation exposure for sun-induced cancers, such as cutaneous squamous cell cancers, and exposure to tobacco smoke carcinogens for smoking-related airway cancers. The epidemiological evidence for both has long been established, although its popular acceptance took some time [145]. Mechanisms of mutation as a result of each exposure have been identified: misrepair by transcription-coupled nucleotide excision repair of UV-induced pyrimidine photodimers [146] and misrepair of guanine damage by the same mechanism [147], respectively. These specific mutational types can now be detected across the genome as mutational signatures [70, 148], and this allows estimation of the contributions of each mutational signature (and potentially the level of mutagen exposure) in any individual tumor [149].

The accrual of mutations over time can now be explored retrospectively in a whole-genome-sequenced tumor. Nik-Zainal et al. [13] used a mutation timing approach to study changes in mutational processes over the life history of breast cancers. By leveraging the power of a large cohort of tumor samples, it becomes possible to identify mutational processes that act early or late in tumor evolution. In lung cancer, the proportion of mutations bearing a smoking signature declines later in tumor evolution, despite ongoing smoke exposure [150, 151]. Conversely, mutagenesis that is related to the activity of the APOBEC family of cytidine deaminases increases later in lung tumor evolution. As expected, inherited defects in DNA repair, such as the deficient mismatch repair seen in Lynch syndrome, can lead to steady and ongoing mutation throughout a tumor’s lifetime [152].

Many mutational signatures do not have identified etiologies, but direct genomic evidence can provide an objective starting point for both epidemiological and biological study. Identifying causative environmental exposures may suggest preventative measures, akin to smoking cessation and UV protection.

### The challenge of somatic variation in normal tissues

The challenge in identifying mutations that are acquired early in tumorigenesis is that many canonical driver mutations, which are thought to be specific and relevant to cancer, may also occur in populations of phenotypically normal cells (Table 1).

Martincorena et al. [77, 129] identified multiple clonal expansions of cells, containing mutations in *TP53*, *NOTCH1*, and other known cancer genes, in both sun-exposed normal eyelids and in aging normal esophagus. Interestingly, mutations were much more common in *NOTCH1* than in *TP53* in normal esophagus, the inverse of the pattern seen in esophageal cancer, suggesting that early *NOTCH1* mutations may protect against cancer development. Demeulemeester et al. [130] analyzed epithelial cells found in bone marrow aspirates of breast cancer patients, identifying cells with copy number aberrations that were completely distinct from the primary breast cancer and therefore from an unknown origin. Gao et al. [153] also detected similar aberrant cells in tissue adjacent to breast tumors that were once again unrelated to tumor cells. Finally, clonal expansions of hematopoietic cells containing leukemia-associated mutations are reported in the circulation of otherwise healthy adults [154, 155]. These confer an increased risk of the subsequent development of a hematological malignancy, but clearly many do not progress [156, 157].

In order to truly reveal the early evolution of cancer, we will need to understand the frequency of these mutational events in the normal tissues in which cancers arise. Cataloguing mutational events in normal tissues, at rare frequencies, will help to identify the cells of origin of cancer as well as the early mutational steps that occur in these cells [158].

## Conclusions and future perspectives

Intra-tumor heterogeneity and the ability of cancers to evolve continuously has proved a major challenge to the implementation of precision anti-cancer medicine. Molecular therapies, predicted to be effective on the basis of the presence of a sensitizing mutation in a single sample, may be of limited clinical benefit. Driver mutations may be subclonal and resistance mechanisms can evolve rapidly [31, 89]. Deeper understanding of this complexity will allow the development of more robust therapeutic strategies. Without doubt, the complexity of tumor evolution is still far from being fully understood, and on an individual basis, tumors will always make unanticipated moves to evade even our best efforts. The recognition that cancer is an evolving system offers a framework on which to hang our clinical and research observations of cancer behavior and biology. We have discussed the more immediate opportunities for translating knowledge of tumor evolution here, but it seems likely that deeper insight will open additional unforeseen avenues.

Insight into the full spectrum of evolutionary paths that cancers can take may lead to the stratification of subsets of cancers that follow specific evolutionary paths. Potentially, the earliest steps or the rate-limiting steps in tumor evolution could be interrupted, either by the identification of preventable etiological factors or by timely medical interventions. These strategies may lead to a significant reduction in the incidence of some cancers or to a high cure rate in early diagnosed cancers, respectively. In addition, once diagnosed, treatment pathways may be matched according to the anticipated evolutionary path of the cancer, as opposed to classification based on traditional histological tumor subtyping. Patients with indolent tumors may be spared therapy altogether. As future therapies emerge, insight into tumor evolution is likely to inform their further development and maximize their impact. Immune checkpoint blockade is possibly the first class of therapy to emerge in this context, reaping the reward of a better understanding of the spectrum of clinical response [92, 94, 159]. Many cancers will probably need an armory of affordable, effective, and tolerable therapies that can be used safely in combination and sequentially. It is likely that conventional therapies—surgery, radiotherapy, and cytotoxic chemotherapy—will continue to have crucial roles in these treatment paradigms, but with a better understanding of the disease, these conventional therapies could be rationally combined with approaches informed by (epi) genomic insights into tumor evolution to achieve improved outcomes for cancer patients.

Box 1 **Glossary**

**Table:** 

Clone	A group of cells that are all descended from a single ancestor. Mutations that are shared between these cells are commonly described as ‘clonal’.
Subclone	Cells originating from a more recent cell than the most recent common ancestor. These will possess both the clonal mutations and also subclonal mutations that are private to the subclone.
Driver mutation	A mutation with a beneficial functional impact on a cell (for example, affecting growth, invasion, or metastasis).
Passenger mutation	A mutation with no functional impact. Both driver and passenger mutations (the latter representing the large majority of mutations) can still be used to identify clonal or subclonal populations.
Most recent common ancestor (MRCA)	The theoretical founder cell of the tumor, from which all cancer cells in a cancer sample are derived. The most recent common ancestor possesses all mutations that are common to all of the tumor cells.
Branching evolution	Divergence in tumor evolution leading to separate subclonal populations.
Linear evolution	The absence of apparent divergence or branches in evolution. All evolution prior to the MRCA will always appear linear as all other pre-MRCA branches have become extinct.
Gradual evolution	An iterative pattern of mutation acquisition and selection over time.
Punctuated evolution	Discontinuous acquisition of mutations over time with periods of relative stasis. Mutations may be acquired in distinct patterns and be co-located, or can be distributed across the genome.

## Abbreviations

CTC: Circulating tumor cell
ctDNA: Circulating tumor DNA
MCRA: Most recent common ancestor
PSA: Prostate-specific antigen
